# Development of a small panel of SNPs to infer ancestry in Chileans that distinguishes Aymara and Mapuche components

**DOI:** 10.1186/s40659-020-00284-5

**Published:** 2020-04-16

**Authors:** Ricardo A. Verdugo, Alex Di Genova, Luisa Herrera, Mauricio Moraga, Mónica Acuña, Soledad Berríos, Elena Llop, Carlos Y. Valenzuela, M. Leonor Bustamante, Dayhana Digman, Adriana Symon, Soledad Asenjo, Pamela López, Alejandro Blanco, José Suazo, Emmanuelle Barozet, Fresia Caba, Marcelo Villalón, Sergio Alvarado, Dante Cáceres, Katherine Salgado, Pilar Portales, Andrés Moreno-Estrada, Christopher R. Gignoux, Karla Sandoval, Carlos D. Bustamante, Celeste Eng, Scott Huntsman, Esteban G. Burchard, Nicolás Loira, Alejandro Maass, Lucía Cifuentes

**Affiliations:** 1 Programa de Genética Humana del ICBM, Facultad de Medicina, Universidad de Chile, Independencia 1027, Santiago, Chile; 2 Departamento de Oncología Básico Clínica, Facultad de Medicina, Universidad de Chile, Santiago, Chile; 3 Mathomics, Centro de Modelamiento Matemático y Centro para la Regulación del Genoma, Facultad de Ciencias Físicas y Matemáticas, Universidad de Chile, Santiago, Chile; 4 Departamento de Psiquiatría, y Salud Mental Norte, Facultad de Medicina, Universidad de Chile, Santiago, Chile; 5 Instituto de Investigación en Ciencias Odontológicas, Facultad de Odontología, Universidad de Chile, Santiago, Chile; 6 Departamento de Sociología, Facultad de Ciencias Sociales, Universidad de Chile, Centro de Estudios de Conflicto y Cohesión, Social, Santiago, Chile; 7 Facultad de Ciencias de la Salud, Universidad de Tarapacá, Arica, Chile; 8 Instituto de Salud Poblacional “Escuela de Salud Pública”, Universidad de Chile, Santiago, Chile; 9 Corporación Municipal de Desarrollo Social, Iquique, Chile; 10 National Laboratory of Genomics for Biodiversity (LANGEBIO), CINVESTAV, Irapuato, Guanajuato, 36821 Mexico; 11 Department of Genetics, Stanford University, Stanford, Palo Alto, CA USA; 12 Department of Medicine, University of California, San Francisco, CA USA; 13 Department of Bioengineering and Therapeutic Sciences, University of California, San Francisco, CA USA; 14 Departamento de Ingeniería Matemática, Facultad de Ciencias Físicas y Matemáticas, Universidad de Chile, Santiago, Chile

**Keywords:** Chile, Admixture, Ancestry, Aymara, Mapuche, SNPs panel

## Abstract

**Background:**

Current South American populations trace their origins mainly to three continental ancestries, i.e. European, Amerindian and African. Individual variation in relative proportions of each of these ancestries may be confounded with socio-economic factors due to population stratification. Therefore, ancestry is a potential confounder variable that should be considered in epidemiologic studies and in public health plans. However, there are few studies that have assessed the ancestry of the current admixed Chilean population. This is partly due to the high cost of genome-scale technologies commonly used to estimate ancestry. In this study we have designed a small panel of SNPs to accurately assess ancestry in the largest sampling to date of the Chilean mestizo population (n = 3349) from eight cities. Our panel is also able to distinguish between the two main Amerindian components of Chileans: Aymara from the north and Mapuche from the south.

**Results:**

A panel of 150 ancestry-informative markers (AIMs) of SNP type was selected to maximize ancestry informativeness and genome coverage. Of these, 147 were successfully genotyped by KASPar assays in 2843 samples, with an average missing rate of 0.012, and a 0.95 concordance with microarray data. The ancestries estimated with the panel of AIMs had relative high correlations (0.88 for European, 0.91 for Amerindian, 0.70 for Aymara, and 0.68 for Mapuche components) with those obtained with AXIOM LAT1 array. The country’s average ancestry was 0.53 ± 0.14 European, 0.04 ± 0.04 African, and 0.42 ± 0.14 Amerindian, disaggregated into 0.18 ± 0.15 Aymara and 0.25 ± 0.13 Mapuche. However, Mapuche ancestry was highest in the south (40.03%) and Aymara in the north (35.61%) as expected from the historical location of these ethnic groups. We make our results available through an online app and demonstrate how it can be used to adjust for ancestry when testing association between incidence of a disease and nongenetic risk factors.

**Conclusions:**

We have conducted the most extensive sampling, across many different cities, of current Chilean population. Ancestry varied significantly by latitude and human development. The panel of AIMs is available to the community for estimating ancestry at low cost in Chileans and other populations with similar ancestry.

## Background

The estimation of ancestral components of current mestizo populations has been the objective of numerous studies in recent years. Knowledge of population ancestry is relevant as a variable to be controlled in genotype-illness association studies [1–3], and is also relevant in forensic genetics [4], prediction of population risks to diseases [5], evolutionary history [6, 7] among other areas. Latin American populations are essentially mixtures from three continents, Europe, America and Africa; the latter being less prevalent in the southernmost countries of South America. The genetic variation in Latin American populations of the southern extreme of South America, including Chile, is underrepresented in publicly accessible world databases. The Chilean population was formed mainly by the mixture of the Amerindian people resident in the territory with the Spanish conquistadors in the middle of the XVI century [8]. The Spanish later brought African slaves; however, their input to the gene pool of the current Chilean population is very small. The native populations that inhabited America before the arrival of European colonists were quite heterogeneous [9]. There were many Amerindian groups present in the territory today known as Chile, with different cultures, languages, social organizations and inhabiting widely different climates and topographies. History indicates that the greatest miscegenation occurred with Aymara in the north and Mapuche in central and southern territories; however, only one recent study has explored the relative contribution of Aymara and Mapuche peoples in the admixture process using genomic approaches [10]. The magnitude of the Amerindian component in the current Chilean population, estimated using blood groups, varies according to socioeconomic strata; ranging from 0% in the highest class to 40% in the lowest class [11]. The estimates on the Chilean population were based mostly on studies of a few urban centers and very few regions of the autosomal genome. Studies using genetic markers with uniparental inheritance have shown an asymmetrical admixture of the ancestral European and Amerindian genomes by sex, as a consequence of Spanish men mating with Amerindian women during the first century of colonization, which has produced 84% Amerindian mitochondrial DNA [12] and less than 20% Amerindian Y chromosomes in the current Chilean population [13, 14]. This asymmetrical admixture has also been demonstrated for other contemporary Latin American populations [15, 16]. In recent years there have been two studies of continental ancestry using SNPs, one of them based on 30 ancestry-informative SNPs [17] and the other, genotyped with the Affymetrix GeneChip Array [18]. Both studies reported that Chileans, in average, have about 43% Amerindian, 55% European and 2% African ancestry [17, 18]. These values are in good agreement with a previous study based on a few autosomal markers [11].

The best estimations of ancestry are obtained by genotyping thousands of SNPs in the whole genome; however, this strategy is costly if many samples are analyzed, while studying too few ancestry-informative markers (AIMs) may produce estimations with little confidence. Thus, small panels of AIMs (from a few dozen to a few hundred SNPs) have been designed, which provide enough information to make ancestry inferences in different human populations [1, 17, 19, 20]. It has been shown that there is a strong correlation between ancestry genetic estimates obtained with sets of AIMs and those obtained with high-density data in samples of Latin America [17]. It has been proposed that panels of 100 SNPs provide reliable estimations of continental ancestry [21]. This technique has been used for some populations in southern South America; for instance, in Argentina using 99, 446 and 46 AIMs [16, 22, 23] and one study in Chile using a panel of 30 AIMs [17]. The aim of the present study was to design and test a panel of AIMs especially designed for the Chilean mestizo population, which is small enough to maintain genotyping costs low, but with enough number of SNPs to allow differentiating the Amerindian ancestries from the north (Aymara) and south (Mapuche) of the country. This panel was designed as part of the ChileGenomico Project, an initiative financed by public funds (Fund for the Promotion of Scientific and Technological Development, FONDEF) and sponsored by the Ministry of Health [24, 25], which involved the largest sample to date of the average Chilean Continental population (n = 3349), including individuals from eight cities spanning 2600 km from north to south. This allowed estimating the magnitude of the ancestral components in the genome of the contemporaneous Chilean population.

## Methods

### Recruitment of participants

**Fig. 1:**
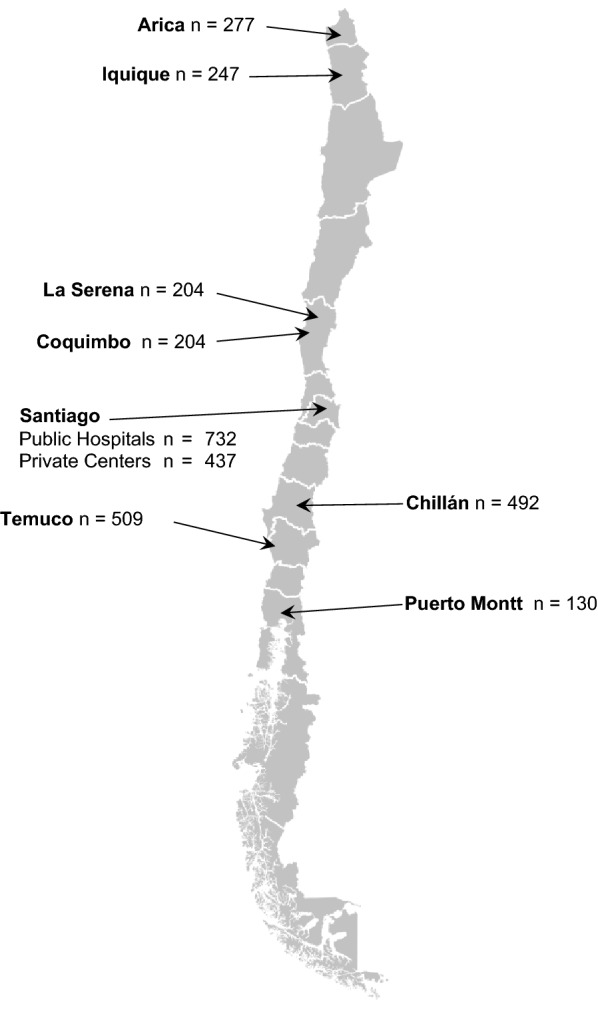
Unrelated participants in ChileGenomico Project by geographic location

Participants of the ChileGenomico Project were recruited from the general Chilean population at regular or temporary blood donation centers in eight cities (Fig. 1). We also included 40 father-mother-offspring trios, who were contacted directly by project researchers. In three trios, DNA extraction failed for one member and only parents were used in this work. Thus, 117 samples from trios were added, making a total of 3349 samples available for this study. We invited the participation of individuals born in Chile over 18 years of age, who declared a permanent address in the country and whose parents were also born in Chile. All signed a document of informed consent. The participation consisted of using 5 ml of the blood they had donated to the Blood Center (transferred to tubes with EDTA) or 3 ml of saliva placed in Oragene Collection kit tubes (DNAGenotek Inc.,Ontario Canada), and answering a questionnaire about their demographic and social background. The protocols and the informed consent questionnaire were reviewed and approved by the corresponding ethics committees of healthcare centers and the ethics committee of the Faculty of Medicine of the Universidad de Chile.

### Extraction of DNA from blood and saliva samples

Five ml of blood samples were collected in tubes containing EDTA. Total DNA was prepared from peripheral blood lymphocytes using the method described by Lahiri & Nurnberger [26]. For trio samples, DNA was extracted from stabilized saliva using the manufacturer’s protocol. Genomic DNA was re-purified using a DNeasy blood and tissue kit, according to the manufacturer’s instructions (QIAGEN Inc., Valencia, California, USA). Genomic DNA integrity was assessed by electrophoresis in 1% agarose gels containing ethidium bromide. All DNA samples were quantified using the Quant-iT™ PicoGreen^®^ dsDNA Assay Kit (Life Technologies Corporation, Carlsbad, CA, USA).

### Chilean Amerindian reference dataset

As reference for Chilean Amerindian ancestry, we selected participants with putative Native American ancestry from two regions of Chile that are located 1750 km apart: from the northern Region of Arica and Parinacota where Aymara (AYM) ancestry is prevalent and from the Metropolitan Region of Chile where Mapuche (MAP) ancestry is more common. In order to be selected, participants had to meet at least two of the following criteria: (1) Self-reporting either having at least one ancestor belonging to an Mapuche, Aymara or Quechua group, or self-identifying with those ethnicities (2) one or more surnames of indigenous origin, (3) mitochondrial haplogroup A, B, C or D, (4) Y Native American chromosome. Seventeen individuals from AYM and 31 individuals from MAP met 2 out of 4 selection criteria, half (27 of 48) were male. Most of the selected individuals (42 of 48) complied with at least three of the aforementioned criteria. Most had at least one indigenous last name (43 of 48). In addition to this, most also had DNAmt of Amerindian origin (43 of 48) and between males, most (14 out of 27) had Y Amerindian chromosome and origin.

DNA from the 48 selected individuals was submitted to the Genomics Core Facility at UCSF for genotyping. DNA was hybridized to Axiom^®^ LAT122 arrays (World Array 4, Affymetrix, Santa Clara, CA). After removing SNPs with < 95% call rate or deviation from Hardy–Weinberg equilibrium (p < 10-6) within each population (CL13 and CL15), 680,397 SNPs remained for the analysis.

### Selection of AIMs

We designed a small panel of 150 SNPs with the objective of determining the percentages of origin of Chilean genomes from four possible ancestries: European, African, Amerindian from northern Chile (Aymara) and Amerindian from southern Chile (Mapuche). This panel included 150 known SNPs, 143 of which included in the AXIOM LATIN1 platform and 7 SNPs detected by sequencing in individuals with high Amerindian ancestry (data available at http://genoma.med.uchile.cl:81/chilegenomico/). Chromosomes X and Y and MtDNA were excluded.

We selected the SNPs to be included in the panel by how ancestry-informative they were, looking for a set of markers that would maximize the differentiation of the four groups mentioned above. As reference we used data of 30 Europeans, 30 Africans from the 1000 Genomes project, 17 Chileans with Aymara ancestry and 31 Chileans with Mapuche ancestry generated in the ChileGenomico project with the AXIOM LATIN1 microarray. Genetic differentiation was estimated using the statistic *I*_*n*_ [27]. We used a genetic algorithm that maximizes simultaneously the *I*_*n*_ of all comparisons as well as genome coverage. We performed numerous trials varying each parameter of the algorithm and selected the one that maximized the correlation between the generated ancestry estimations and those obtained with the complete set of SNPs present in the microarray. Ancestry estimations were performed by comparing the allele frequencies of the SNPs obtained in the ChileGenomico Project with those of the reference population using the ADMIXTURE program [28].

### Genotyping with the AIMs panel

We genotyped the remaining Chileans with the panel of 150 ancestry-informative SNPs designed (CLG) in LGC Limited, Middlesex (UK), and inferred the European, African, northern and southern Chile Amerindians using the ADMIXTURE program. Three samples were genotyped in duplicate as a control.

### Quality control of the panel of AIMs

Precision of panel was assessed by the correlation between the ADMIXTURE inferences of ancestry obtained with our panel of AIMs (CLG) and genome-wide genotypes for a reference dataset containing 30 YRI and 30 CEU from 1000G [REF], 95 individuals with high Amerindian ancestry from southern Chile from the PatagoniaDNA Project [29] and 419 admixed Chileans from the ChileGenomico Project who were previously genotyped with the AXIOM LATIN1 platform (unpublished).

## Results

Additional file 1: Table S1 contains the list of 147 SNPs included in the ChileGenomico panel. It shows their chromosomal location and the minimum allele frequencies observed in the 2843 Chileans genotyped. Genotyping failed for three markers, rs 2009923413, rs62045476 and rn145426 (novel, position 4:100673238); thus, we did not obtain data for them and they are not included. The average of missing genotype rate was 0.012, and the concordance with the microarray data was 0.95. It can be seen that Minor Allele Frequencies or MAF were variable, from 0.003 to 0.5 with a mean of 0.27.

### Comparison with AXIOM LATIN1 data

Some of the CLG panel samples had been genotyped with AXIOM LATIN1 (n = 156). There was a total of 156 samples and 141 SNPs in common between AXIOM LATIN1 and CLG, since there were six AIMs that did not have data in AXIOM LATIN1 (see Methods). Comparing the genotypes obtained with AXIOM LATIN1 and the CLG panel, the concordance was above 0.95 in all samples.

### Validation of the SNPs Panel

**Fig. 2:**
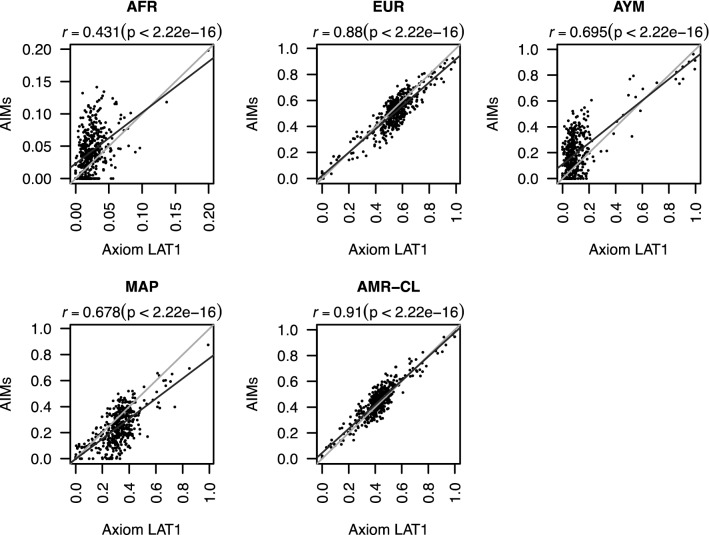
Comparison of ancestry values estimated using data from the microarrays (AXIOM LAT1) and the panel of AIMs in 156 Chilean mestizos. Ancestry was estimated for African (AFR), European (EUR), Aymara (AYM), and Mapuche (MAP) components. AMR-CL represents Chilean Amerindian ancestry estimated as the sum of AYM and MAP

We compared the results of ancestry inference obtained using genotypes from the AXIOM LATIN1 platform with those of our panel of AIMs (CLG panel) using ADMIXTURE in the 156 individuals in both datasets. The analysis with K = 3, assuming three ancestral populations had a correlation of 0.88 for inferences of European ancestry and 0.91 for Amerindian ancestry. Increasing to K = 4 gave a correlation of 0.70 for Aymara ancestry and 0.68 for Mapuche ancestry (Fig. 2). African ancestry had a low correlation (0.43), but different from 0 (p = 7 × 10^−7^), given that this was a very minor component in the participants of ChileGenomico.

### Ancestry inference for 2843 Chileans based on the CLG panel

**Fig. 3:**
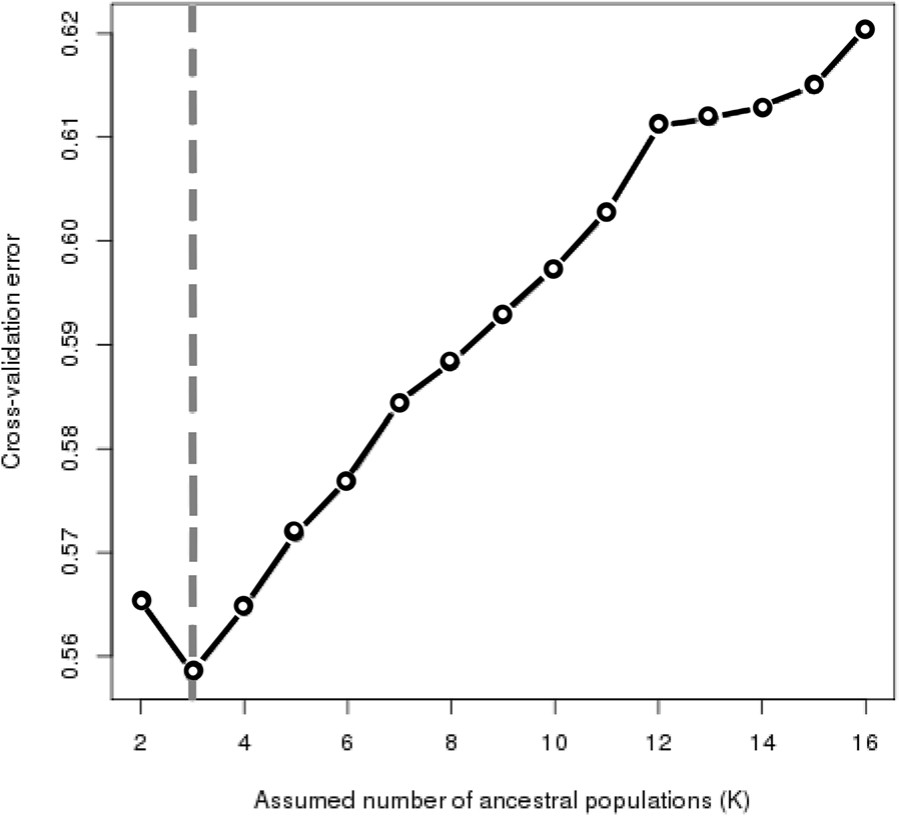
Cross-validation error. The genotype of one-fifth of the markers was predicted using the ancestry estimated in the remaining four-fifths

Using the ADMIXTURE [28] software, we inferred the ancestry components based on the panel designed (CLG panel), with data of 147 SNPs. The reference population data of 30 European and 30 African individuals were obtained from the 1000 Genome project, populations CEU and YRI, respectively. We used 30 Aymara individuals from Peru as reference for Aymara ancestry, and 30 Pehuenches and Huilliches from southern Chile for Mapuche ancestry (CLS) [29, 30]. Four SNPs were physically close, with linkage disequilibrium r^2^ ≥ 0.2; however, as eliminating them did not affect the ancestry results significantly, they were kept in the panel. A cross-validation test suggested K = 3 or K = 4 as the number of ancestry components (Fig. 3).

**Fig. 4:**
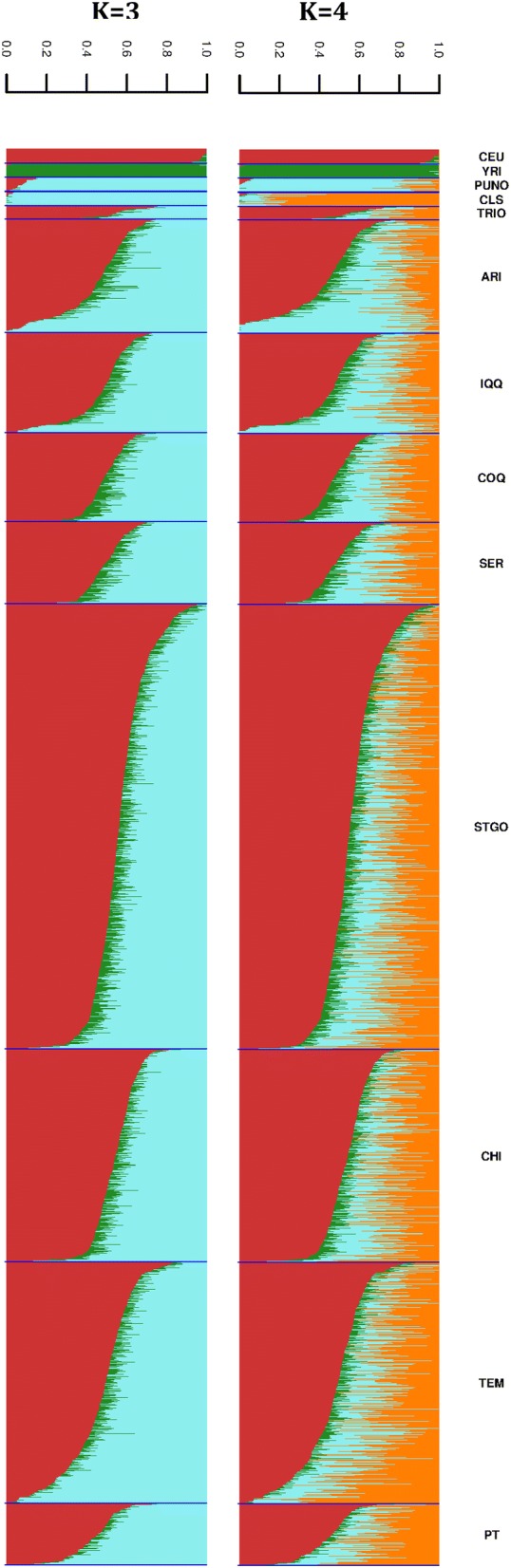
Ancestry inferences in unrelated participants of the ChileGenomico project. Populations CEU and YRI are from the 1000 Genomes project and represent European and African ancestry, respectively. Peruvian Amerindians data was obtained by collaboration with the group of Carlos Bustamante (Stanford University). The CLS individuals have Pehuenche and Huilliche ancestry; they were selected from the Patagonia DNA project for their high Amerindian ancestry

The results of ancestry inference are shown in Fig. 4 and summarized in Table 1. The percentage of European ancestry varied between 0 (in 8 individuals) and 100% (in one individual) in the Chileans studied, same for the percentage of Amerindian ancestry which also varied from 0 (in 4 individuals) to 100% (in 2 individuals). The average Amerindian ancestry was 0.42 ± 0.14, with discrete differences among the areas sampled. However, there was greater heterogeneity for the Aymara and Mapuche components in the country; the former was more important in the north, reaching 36%, than in the center and south, where it was only 16–17%. The Mapuche component was only 18% in the extreme north of the country, while it reached 35% in the south.

**Table 1:** Mean ancestry proportion and standard errors in different cities of Chile using admixture

Sampling city (percentage of country population)	N	European	African	Amerindian	Aymara	Mapuche
Reference populations						
CEU	30	0.97 ± 0.01	0.02 ± 0.00	0.00 ± 0.00	0.01 ± 0.00	0.00 ± 0.00
YRI	30	0.00 ± 0.00	0.99 ± 0.00	0.00 ± 0.00	0.00 ± 0.00	0.00 ± 0.00
PUNO	30	0.02 ± 0.00	0.00 ± 0.00	0.92 ± 0.01	0.83 ± 0.02	0.15 ± 0.02
CLS	30	0.01 ± 0.01	0.01 ± 0.00	0.98 ± 0.01	0.16 ± 0.01	0.82 ± 0.02
ChileGenomico samples*						
Arica (1.3)	239	0.41 ± 0.01	0.05 ± 0.00	0.50 ± 0.01	0.36 ± 0.02	0.18 ± 0.01
Iquique (1.9)	211	0.45 ± 0.01	0.05 ± 0.00	0.47 ± 0.01	0.30 ± 0.01	0.20 ± 0.01
Serena (1.2)	172	0.48 ± 0.01	0.05 ± 0.00	0.45 ± 0.01	0.24 ± 0.01	0.23 ± 0.01
Coquimbo (3.1)	182	0.48 ± 0.01	0.06 ± 0.00	0.45 ± 0.01	0.24 ± 0.01	0.22 ± 0.01
Santiago public hospitals (28.3)	498	0.53 ± 0.01	0.04 ± 0.00	0.42 ± 0.00	0.18 ± 0.01	0.25 ± 0.01
Santiago private centers (12.14)	432	0.60 ± 0.01	0.04 ± 0.00	0.34 ± 0.01	0.16 ± 0.01	0.20 ± 0.01
Chillán (8.9)	442	0.54 ± 0.00	0.04 ± 0.00	0.42 ± 0.00	0.17 ± 0.01	0.25 ± 0.01
Temuco (5.5)	509	0.47 ± 0.01	0.04 ± 0.00	0.49 ± 0.01	0.18 ± 0.01	0.31 ± 0.01
Puerto Montt (4.7)	130	0.46 ± 0.01	0.03 ± 0.00	0.52 ± 0.01	0.17 ± 0.01	0.35 ± 0.01
TRIOS	28	0.57 ± 0.02	0.04 ± 0.01	0.38 ± 0.02	0.18 ± 0.02	0.21 ± 0.02
Total ChileGenomico**	2843	0.53 ± 0.14	0.04 ± 0.04	0.42 ± 0.14	0.18 ± 0.15	0.25 ± 0.13

All values were estimated with K = 4, except the Amerindian ancestry in the central column which used K = 3

* ChileGenomico Project **The total percentages of ancestry were calculated by a weighted average according to the fraction of the population that lives in each of the cities

### Variation in the magnitude of the Amerindian component

**Fig. 5:**
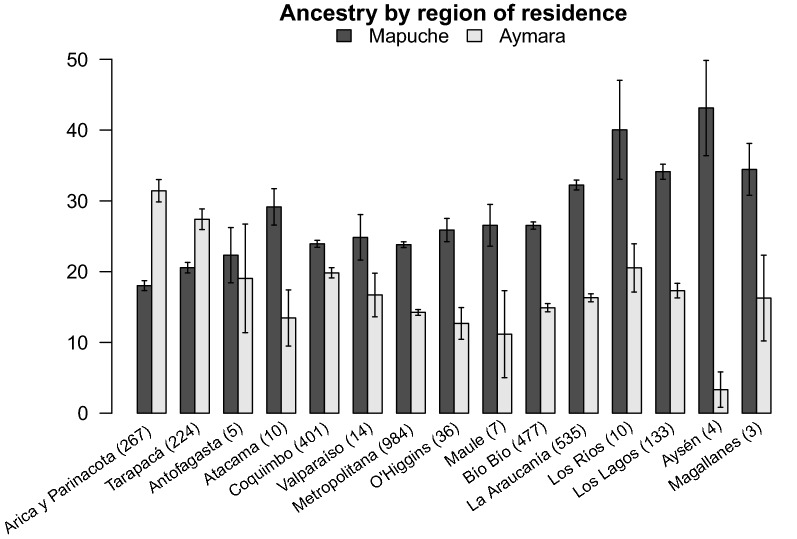
Percentages of Mapuche (white) and Aymara (black) ancestry estimated with a panel of 147 informative SNPs according to region of residence in Chile, ordered from north to south (p < 0.001)

The highest percentages of Amerindian ancestry were found in individuals sampled in the most extreme regions: Arica in the north and Temuco and Puerto Montt in the south and were lowest in the private centers of Santiago. The level of Amerindian ancestry is also different according to the region of residence of the individuals, although very few individuals were recruited from some regions (Fig. 5). The highest percentages of mean Amerindian ancestry were found in the Chileans residing in the southernmost cities sampled, while the lowest mean levels were found in the residents in the center of the country (between Valparaíso and Bío, see Fig. 1). The average Aymara and Mapuche ancestries in Chileans were 18% and 25%, respectively. However, these values were variable throughout the country; the first was especially high in the north (40.03%) and gradually decreased southwards, while the opposite occurred with the Mapuche ancestry reaching 35.61% in the south (Fig. 5).

**Fig. 6:**
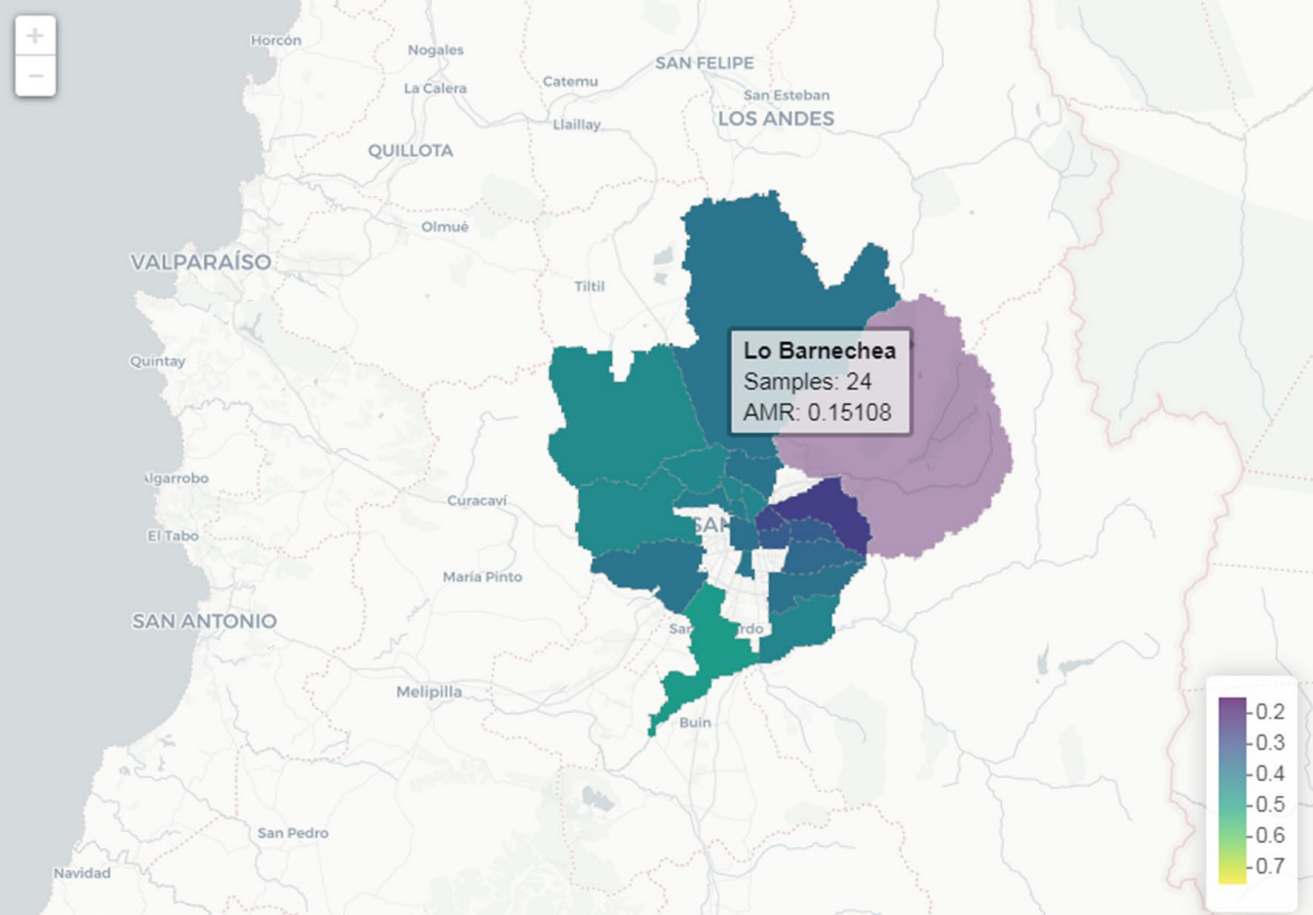
Percentages of Amerindian-ancestry-estimated commune in the Metropolitan Region of Chile

Additionally, we built an on line platform where ancestry data can be displayed by communes (they refer to the most basic administrative division in Chilean cities), by any user (http://genoma.med.uchile.cl/ancestry/) (Fig. 6).

### Relevance of ancestry for population health

**Fig. 7:**
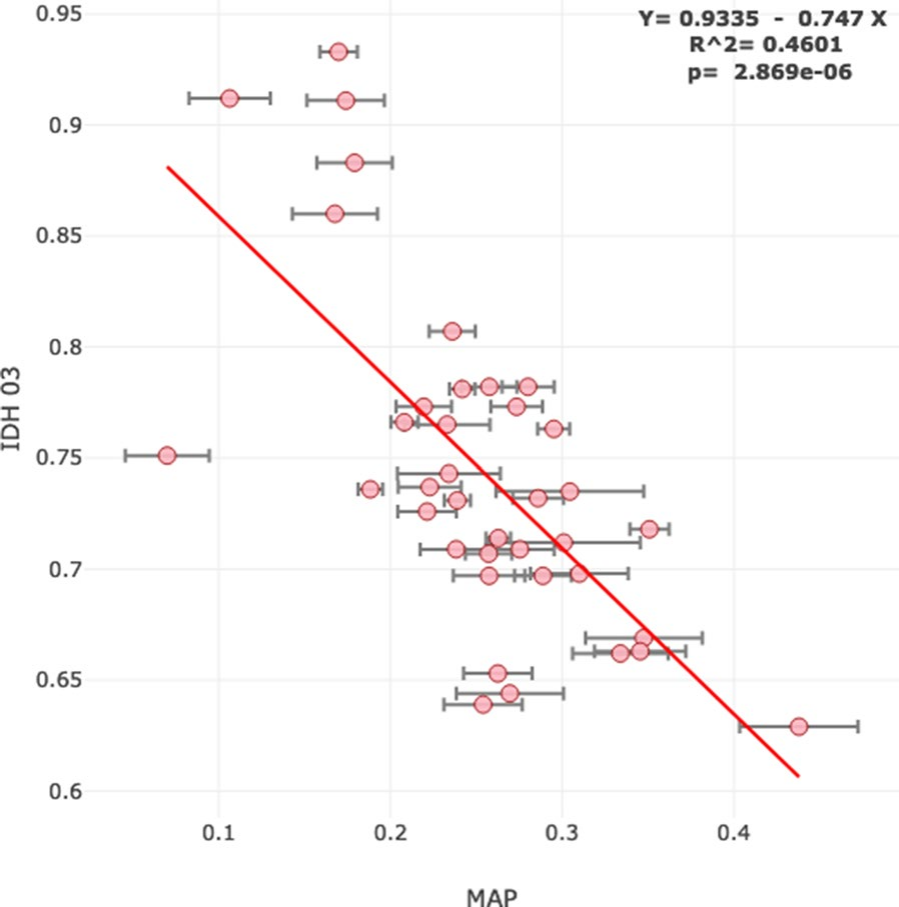
Human Development Index of 40 communes of Chile by average Mapuche ancestry. Graph created by ChileGenomico’s shiny app

In order to test association between ancestry and quality of life, we compared average Amerindian ancestry per commune of Chile with the Human Development Index (HDI), constructed by the Chilean Ministry of Planning and the United Nations Development Programme (UNDP), which was used to explain socioeconomic differences among districts [31]. We observed a significant negative correlation between Amerindian ancestry and HDI, where the expected decrease from a commune with 0% to a commune with 100% Amerindian ancestry was of 0.747, in a theoretical range that goes from 0 (lowest) to 1 (highest) development (Fig. 7). These HDI values are available as a csv file downloadable from the ChileGenomico’s Shiny app at http://genoma.med.uchile.cl/ancestry/. This HDI summarizes three components: access to health, access to education, and income. The linear and negative association that we observed between Amerindian ancestry and HDI shows evidence of a genetic structure that is associated to quality of life.

**Fig. 8:**
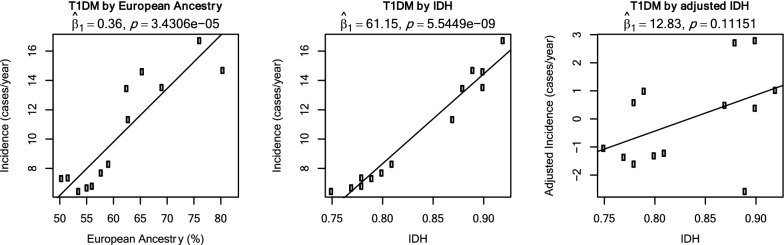
Incidence of Type 1 Diabetes Mellitus by European Ancestry (left), Human Development Index (HDI, middle), and IDH after adjusting the incidence by European ancestry. Only communes of the Metropolitan Region of Chile are shown

With the purpose of demonstrating one potential application of our results, we re-examined the hypothesis of the role of HDI on incidence of Type 1 Diabetes Mellitus that was previously proposed. By analyzing the geographic clustering of HDI with incidence in the Metropolitan region of Santiago, Torres-Avilés et al. [32] proposed that socioeconomic variables were involved in the risk of diseases. However, given the high level of genetic structuration that we observed in the population, socioeconomic factor may be confounded with genetic ones. Therefore, we tested their hypothesis but accounting for ancestry. A linear model of incidence by commune on both IDH and Amerindian ancestry showed that HDI remained highly significant (p < 2.55e−05). If one adjusts first the incidence by ancestry and then regresses it on HDI, significance is reduced; but the trend it is still evident (Fig. 8). Thus, genetic structuration of the population cannot account for the association observed by Torres-Avilés et al. (2010) and other variables influencing HDI must be involved. This type of analysis can be easily performed using ChileGenomico’s app and the HDI data is provided as an example of an external dataset that the user can upload to be analyzed in conjunction with our ancestry estimated per commune of Chile.

## Discussion

In this study we developed a small panel of 147 autosomal SNPs appropriate to infer continental ancestry and capable of distinguishing between the Amerindian components of northern and southern Chile. We validated the ancestry estimations obtained with this panel, comparing them with the inferences performed with high-density genotyping with the AXIOM LATIN1 platform. We found correlations of 0.91 and 0.88 for the Amerindian and European components, respectively, which indicates the reliability of the panel to infer these continental components. Other similar initiatives to infer continental ancestry in the Latin American population have been published, such as the panel described by Avena et al. [22] using 99 AIMs and another by Ruiz-Linares et al. [17] using 30 AIMs; the Amerindian ancestry percentages inferred with these panels had correlations of 0.93 and 0.70, respectively, with respect to high-depth genotyping (more than 50,000 SNPs). Ours is the first panel of AIMs that allows distinguishing between Aymara and Mapuche components for populations of southern South America; the set of SNPs we designed allows inferring Aymara and Mapuche ancestry with reliability similar to that obtained with thousands of genotypes in the AXIOM LATIN1 platform (correlations of 0.70 and 0.68, respectively). The inference of African ancestry that we obtained with a panel of 147 SNPs had a low correlation (0.39) with the AXIOM LATIN1 genotype estimation, due to the low percentage of the African component in the Chilean population. The variance of this estimation is large, so the Africans who came to Chile may not be representative of the genotypes present in the current databases. For these reasons our panel is especially recommended for populations of the south of Latin America with a low African component.

By studying a large sample of contemporary Chilean mestizos of the largest urban centers of the country, we obtained continental ancestry inferences concordant with those published for this population [11, 18, 33].

The Amerindian component in the country is higher in the north and south and lower in the center, which may be because the current descendants of the original populations remain mostly in these regions, while the majority of the European immigration in the last few centuries came directly to the central area of the country, or if they first came to other regions, migrated to the capital as part of the rural–urban movement characteristic of this epoch. The Aymara component is especially high in the north and gradually decreases southwards; the opposite occurs with the Mapuche ancestry, in line with the historical locations of these native people.

The African component of the Chilean population is low; the slaves who arrived in the north with the Spanish conquerors were not followed by new African immigration, while European immigrants continued to arrive. This may change in the next century, since in recent years Chile has been receiving immigrants from Haiti and the Dominican Republic, and from countries of northern South America where there is a large African component.

One limitation of our study is the fact of having sampled individuals in blood banks of the mayor urban centers instead of having a probabilistic sampling method that faithfully represents all of contemporary Chilean population. This means that our results reflect an approximation of what happens in Chilean mixed population in the sampled cities and does not represent the whole genetic variability present in the rural regions of the country, the extreme South (further than Puerto Montt) and from insular territories. Nevertheless, our estimations are trustworthy as an approximation of a high proportion of urban Chileans in the cities included in the sampling, considering that the individuals present in the sample were not selected for having any particular condition (such as having a specific illnesses, residents of only one city, or belonging to any particular institution). Another limitation is the fact of having studied only Mapuche and Aymara referents for the Amerindian component of Chileans, knowing that there were other indigenous groups that contributed to the general genetic makeup. Unfortunately, we don’t have contemporary referents for those ethnicities; such is the case for *atacameños, diaguitas, picunches,* etc. Nonetheless, Mapuche and Aymaras are the Amerindian groups which contributed the most to the genetic composition of contemporary Chileans, thus in this research we are capturing the larger part of this component.

Even though the number of individuals considered as ancestral referents (30, 30, 41 and 17 for European, African, Mapuche and Aymara ancestry, respectively) is not too high, it allowed us to obtain trustworthy estimations which are in line with all other ancestry estimations obtained in studies of the Chilean population through genome-wide data analysis, including both continental ancestry [17, 18] and, Mapuche and Aymara ancestry [10].

In summary, the panel we have designed allowed us to differentiate the Aymara and Mapuche components in the current mestizo population, the two main components of the Amerindian ancestry of Chileans. This distinction is important from an historical or sociological point of view, but also is relevant to identify the specific ancestry that is associated with a higher risk for certain diseases, which have been reported as more prevalent in populations with greater Amerindian ancestry; such is the case of the known association of cholelithiasis and gallbladder cancer with Native American ancestry, but only recently it has been shown that it is the Mapuche ancestry that is associated with the greater risk of gallbladder cancer and not the Aymara component [10, 34].

Finally, we make our results available through an online app and demonstrate how it can be used to adjust for ancestry when testing association between incidence of a disease and non-genetic risk factors.

## Conclusions

The designed SNPs panel is able to distinguish between the two main Amerindian components in the current Chilean mixed population: Aymara in the North and Mapuche in the South. The Chilean population has an average of 42% Amerindian ancestry, which varies according to geographic region and socioeconomic stratum. The average components of Aymara and Mapuche ancestry are 18 and 25%, respectively. Aymara ancestry is higher in the north of the country and decreases towards the south, while the opposite occurs with Mapuche ancestry. The SNPs panel implemented here can be used at a fraction of the cost of microarrays, thus providing an additional tool for the research community. Our panel could be used for population genetic studies interested in the ancestry of Chileans and in epidemiologic studies of non-communicable diseases to account for population structure. Researchers interested in assessing ancestry in their studies, can use the list of SNPs provided here to develop their own assays or can request genotyping as a service from the ChileGenomico laboratory through its website.

## Supplementary information

**Additional file 1.** List of the SNPs present in the designed panel (CLG).

## Data Availability

See app at http://genoma.med.uchile.cl/ancestry/, and see unpublished data on individuals with high Amerindian ancestry at http://genoma.med.uchile.cl:81/chilegenomico/.

## Abbreviations

AIM: Ancestry informative marker
CLG: panelsnps panel designed in this article that differentiates Aymara and Mapuche component
CEU: References for European ancestry, from the 1000 genome Project
CLS: Pehuenches and Huilliches from southern Chile, references for Mapuche ancestry
MAF: Minor allele frequency
SNP: Single nucleotide polymorphism
YRI: References for African ancestry, from the 1000 genome Project
